# Supplementary material for: Ethylenediaminetetraacetic Acid Disodium Salt Acts as an Antifungal Candidate Molecule against Fusarium graminearum by Inhibiting DON Biosynthesis and Chitin Synthase Activity
Source: Toxins (Basel). 2020 Dec 27;13(1):17. doi: 10.3390/toxins13010017 (PMC7823441; doi:10.3390/toxins13010017)
Supplement: Supplementary file 1 [file toxins-13-00017-s001.pdf]

Supplementary Materials: Ethylenediaminetetraacetic Acid Disodium Salt Acts as an Antifungal Candidate Molecule against *Fusarium graminearum* by Inhibiting DON Biosynthesis and Chitin Synthase Activity

Xiu-Shi Song, Kai-Xin Gu, Jing Gao, Jian-Xin Wang, Shao-Chen Ding and Mingguo Zhou

Table S1. Minimum inhibitory concentrations of $EDTANa_2$.

Isolates	MIC (mM)
F. acuminatum	18.75
F. asiaticum	9.37
F. avenaceum	9.37
F. concentricum	18.75
F. culmorum	18.75
F. equiseti	9.37
F. fujikuroi	9.37
F. graminearum	18.75
F. lateritium[a]	200
F. oxysporum	18.75
F. proliferatum	9.37
F. solani	18.75
F. verticillioides	18.75

[a] $EDTANa_2$ had no fungicidal activity in the range of 4.69-150 mM. The experiment was replicated three times.

Table S2. Strains used in this study.

Strain	Species	Description
2021	*Fusarium asiaticum*	Isolated from a field experiencing Fusarium head blight epidemic in Jiangsu Province, China
PH-1	*Fusarium graminearum*	*Fusarium* reference strain PH-1
F. acuminatum	*Fusarium acuminatum*	Isolated from a field experiencing soybean Fusarium Root Rot in Liaoning Province, China
F. avenaceum	*Fusarium avenaceum*	Isolated from a root of naked barley in Gansu Province, China
F. concentricum	*Fusarium concentricum*	Provided by Shanghai Academy of Agricultural Sciences
F. culmorum	*Fusarium culmorum*	Provided by Shanghai Academy of Agricultural Sciences
F. equiseti	*Fusarium equiseti*	Isolated from alfalfa root rot disease suspected samples in Neimenggu Province, China
F. fujikuroi	*Fusarium fujikuroi*	Isolated from a field experiencing rice bakanae disease in Jiangsu Province, China

F. lateritium	*Fusarium lateritium*	Isolated from alfalfa root rot disease suspected samples in Neimenggu Province, China
F. oxysporum	*Fusarium oxysporum*	Isolated from alfalfa root rot disease suspected samples in Neimenggu Province, China
F. proliferatum	*Fusarium proliferatum*	Isolated from a field experiencing rice bakanae disease in Jiangsu Province, China
F. solani	*Fusarium solani*	Isolated from a field experiencing soybean Fusarium Root Rot in Liaoning Province, China
F. verticillioides	*Fusarium verticillioides*	Provided by Shanghai Academy of Agricultural Sciences

Table S3. Primers used in *TRI* genes qPCR.

Primers	Sequences
FGSG_00071 (*Tri1*) P1	GAAGATTCCTGAAGGTCCCG
FGSG_00071 (*Tri1*)P2	TGTACCAATTCCAATCGCAGAC
FGSG_02343 (*Tri12*) P1	ATCCCCGCAGTCATTCAGA
FGSG_02343 (*Tri12*) P2	CGCAATCATAGCCACTACACC
FGSG_03532 (*Tri8*) P1	ATTTACCAAGCTATCCACGACG
FGSG_03532 (*Tri8*) P2	CACTGAAAAGCCGCCTCAT
FGSG_03534 (*Tri3*) P1	ACGGTGTCAACGAACGCTT
FGSG_03534 (*Tri3*) P2	TCCAGTCGGATTGCCAGATA
FGSG_03535 (*Tri4*)P1	ATGGCCTTTGCTGAGATGTACC
FGSG_03535 (*Tri4*)P2	CCAACAATACGGGCGTGAGT
FGSG_03536 (*Tri6*) P1	ACCGTGCTCGGCATGAGT
FGSG_03536 (*Tri6*) P2	TCCACCCTGCTAAAGACCCT
FGSG_03537 (*Tri5*)P1	AGCTCACCCAGGAAACCCT
FGSG_03537 (*Tri5*)P2	CACTCAATCGTGTCCATCACC
FGSG_03538 (*Tri10*) P1	TGGGCTCGACAAGACATTT
FGSG_03538 (*Tri10*) P2	CCATCCCTCAACCAAGACA
FGSG_03540 (*Tri11*) P1	TTCACCCGACCAAACGACT
FGSG_03540 (*Tri11*) P2	TCTGCGTAGGCAAGGTTCAT
FGSG_07896 (*Tri101*) P1	CGCCAGCGAACAAGAGGT
FGSG_07896 (*Tri101*) P2	AAAGTCGTAATCCCAGAGTCCC
TubulinP1	CGTCCAGAGCAAGAACTCATCA
TubulinP2	TGCGTCGGAACATAGCAGTAA

	Query Cover	E value	Per. Identity	Accession
F. acuminatum-VS-*F. acuminatum* isolate SYQ2	100%	0.0	100.00%	MT649858.1
F. avenaceum-VS-*F. avenaceum* strain ZMXR13	95%	0.0	99.27%	MT446118.1
F. concentricum-VS-*F. fujikuroi* isolate GX12	96%	0.0	99.63%	KJ000433.1
F. culmonum-VS-*F. culmonum* isolate F3-3-19	99%	0.0	98.73%	KX349468.1
F. equiseti-VS-*F. equiseti* isolate USM3-2A	99%	0.0	99.09%	KM111481.1
F. fujikuroi-VS- *F. fujikuroi* isolate GX12	98%	0.0	99.27%	KJ000433.1
F. lateritium-VS-*F. lateritium* strain ZMT01	97%	0.0	99.45%	MT875260.1
F. oxysporum-VS-*F. oxysporum* strain WZ-198	99%	0.0	99.27%	MN856331.1
F. proliferatum-VS-*F. proliferatum* strain BCD	97%	0.0	99.81%	KU939074.1
***F. solani*-VS-*F. solani* isolate TS08-95-3**	**95%**	**0.0**	**99.09%**	**AB470848.1**
***F. verticillioides*-VS-*F. verticillioides* isolate Q2**	**96%**	**0.0**	**99.26%**	**MN698249.1**

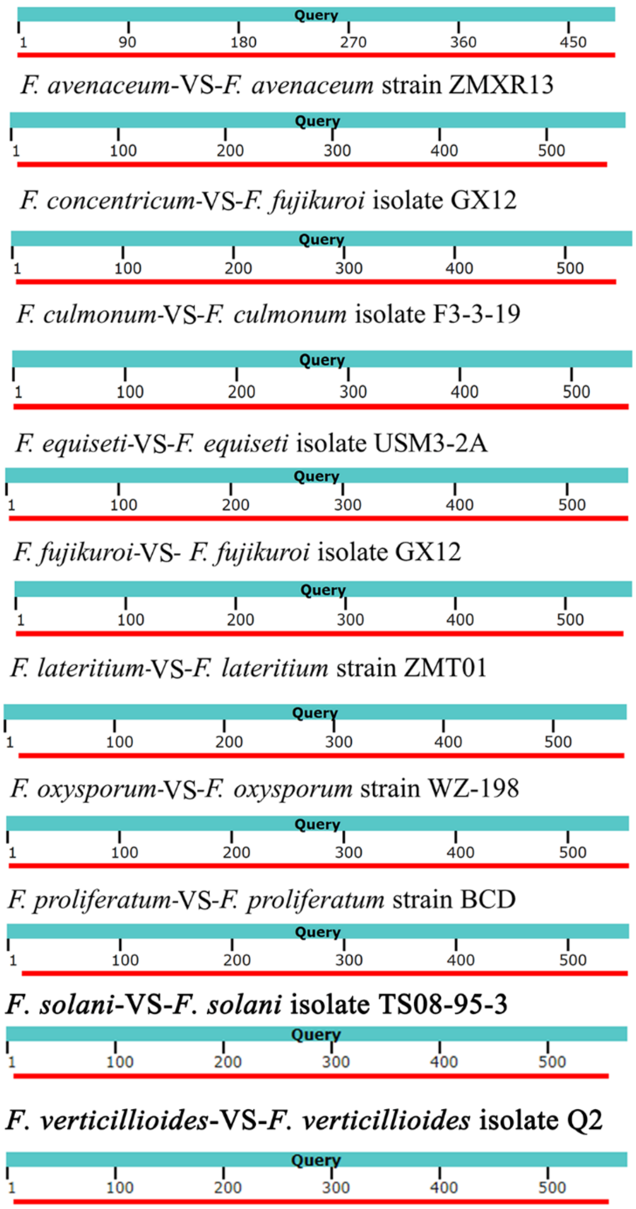

Figure S1. Internal transcribed spacer (ITS) sequences alignment of *Fusarium* isolates used in this study. ITS sequences of different strains were amplified using primers ITS-F/ITS-R (ITS-F: TCCGTAGGTGAACCTGCGG, ITS-R: TCCTCCGCTTATTGATATGC). Sequences alignment was performed using NCBI Blastn (https://blast.ncbi.nlm.nih.gov/Blast.cgi) with default parameters.
